# Cisplatin Ototoxicity: Examination of the Impact of Dosing, Infusion Times, and Schedules In Pediatric Cancer Patients

**DOI:** 10.3389/fonc.2021.673080

**Published:** 2021-06-28

**Authors:** Miranda L. Camet, Anne Spence, Susan S. Hayashi, Ningying Wu, Jennifer Henry, Kara Sauerburger, Robert J. Hayashi

**Affiliations:** ^1^ Division of Pediatric Hematology/Oncology, Washington University School of Medicine, St. Louis, MO, United States; ^2^ Biostatistics Shared Resource, Division of Public Health Sciences, Department of Surgery, The Alvin J. Siteman Comprehensive Cancer Center, Washington University School of Medicine, St. Louis, MO, United States; ^3^ Division of Pediatric Hematology/Oncology, Washington University School of Medicine, St. Louis Children’s Hospital, St. Louis, MO, United States

**Keywords:** cisplatin, pediatric, cancer, ototoxicity, dosing

## Abstract

**Background:**

Sensorineural hearing loss is a well-known side effect of cisplatin (CDDP). There is limited research on the effect of dosing, infusion times, and schedules of cisplatin administration and their impact on hearing loss.

**Methods:**

A retrospective review of 993 pediatric patients’ medical and audiological charts from August 1990 to March 2015 was conducted using stringent inclusion criteria to characterize patients with hearing loss. 248 of these patients received CDDP. Of these, 216 patients had sufficient CDDP infusion data to assess for sensorineural hearing loss attributable to CDDP and its associated risk factors. Chart reviews were performed to extract clinical data including CDDP dosing information. Demographic and clinical characteristics were summarized by descriptive statistics, and univariate and multivariate logistic regressions were performed to examine the relationship between hearing loss and specific parameters of cisplatin administration (amount infused per dose, prescribed infusion time, total number of doses, number of doses per cycle, number of cycles, cumulative cisplatin exposure). Stepwise variable selection procedure was performed in the multivariate model building to extract the best subset of risk factors for the prediction of hearing loss and worsening ototoxicity grade using an established ototoxicity grading scale from the International Society of Pediatric Oncology (SIOP).

**Results:**

A total of 153 patients with complete medical and audiologic data were evaluable for analysis. Hearing loss was identified in 72.6% of the patients. Multivariate analysis revealed that age [OR=0.90 (0.84-0.97), *p*-value=0.0086], radiation to any part of the body, [OR=3.20 (1.29-7.93), *p*-value=0.012], amount infused per dose (mg/m^2^) [OR=1.018 (1.002-1.033), *p*-value=0.029], and cumulative cisplatin exposure (mg/m ^2^) [OR=1.004 (1-1.008), *p*-value=0.027] were associated with hearing loss. Similar associations were also found between these risk factors and worsening SIOP grade.

**Conclusion:**

In one of the largest studies examining the influence of CDDP dosing and schedules on hearing loss, we found the amount of CDDP infused per dose is a significant risk factor. Considerations in designing regimens that reduce the amount of CDDP infused per dose may reduce the risk of hearing loss. Randomized prospective trials are needed.

## Introduction

Sensorineural hearing loss (SNHL) is a well-known complication from the administration of cisplatin (CDDP) (1–6). Evidence suggests there is long-term retention of CDDP in the cochlea, and a dose-dependent relationship between a higher cumulative dose and a higher incidence of hearing loss has been established (7–9). Hearing loss may evolve during therapy, after its completion, or may not even present until years following the end of treatment (5, 10–12). Though the relationship between hearing loss and CDDP has been thoroughly examined, the effect of variables related to CDDP dosing and administration (the amount per dose, frequency, and dosing schedules) and their relationship to hearing loss have not been well-defined. This lack of knowledge limits our ability to establish strategies for reducing ototoxicity through modification of dosing parameters (4, 13, 14). Due to the limited research on this topic to date, we sought to examine the relationship between parameters of CDDP dosage or administration and the presence of hearing loss in a cohort of pediatric cancer survivors.

## Patients and Methods

This study, approved by the Institutional Review Board of the Washington University School of Medicine Human Research Protection Office, was a retrospective chart review of medical record data existing at the initiation of our study. Audiology charts of pediatric oncology patients at St. Louis Children’s Hospital treated from August 1, 1990 through March 31, 2015 were reviewed. From this cohort, patients were assessed for treatment containing CDDP.

Inclusion criteria into our current study required prior CDDP treatment. Patients whose chemotherapy treatment did not include CDDP were excluded. Evaluable patients had either completed their CDDP therapy or had documented CDDP hearing loss from current therapy and were still being treated at the cutoff for study entry in March 2015. Data was not collected for patients still undergoing treatment who had normal hearing at the cutoff point of the study. No patients received oto-protectants. The medical records of these patients were reviewed to extract the following variables of interest: gender, birthdate, date of diagnosis, race, ethnicity, diagnosis, CDDP dosage information (i.e. cumulative dose, number of doses, amount per dose, doses per cycle, dosage time, dosage reduction), presence of carboplatin, radiation exposure to any part of body, radiation exposure to head, date when all therapy ended, date of last CDDP administration, living status, date of most recent audiogram, presence of hearing loss based on the worse ear, and right/left ear toxicity grades according to the International Society of Pediatric Oncology (SIOP) (15). Each patient’s chart was individually evaluated for the specific variables mentioned above; the actual dosing of CDDP recorded in the medical record was used, and there was no imputed data regarding the amount of CDDP the patient received to ensure that the dosage information is patient specific. We were unable to obtain consistent and specific documentation of the infusion times for CDDP. Infusion times utilized were the prescribed infusion times derived from the patient’s orders or treatment plan. After review, thirty-two patients were excluded as specific CDDP dosage information was not available, resulting in a cohort of 216 patients eligible for analysis in the current study.

A substantial effort was made to ensure clear audiologic data. To uphold the audiologic parameters utilized in our previously reported investigations, we adhered to formerly established criterion to ensure the study subjects had treatment acquired, ear specific, sensorineural hearing loss (16). This resulted in the exclusion of many patients but created a pediatric population with less ambiguous hearing profiles and allowed for a more rigorous investigation into the presence of ototoxic hearing loss in this population. The following details the stringent audiologic parameters required for inclusion in this study.

### Baseline Audiograms

All audiograms included in our analysis were of good to fair reliability, as determined by the testing audiologist. Soundfield and ABR testing was allowed for baseline testing. All patients were required to have a normal baseline audiogram with a subsequent ear specific behavioral audiogram, testing out to 6000 and/or 8000 Hz; baseline audiograms obtained *via* soundfield testing were included as long as the subsequent hearing test revealed ear specific thresholds. According to our institutional standards, a normal behavioral hearing test was defined as thresholds of ≤ 20 dB HL from 1 kHz - 4 kHz and ≤ 30 dB HL at 6 kHz and 8 kHz, in order to account for tympanostomy tubes and collapsing canals from earphones. A normal auditory brainstem response (ABR) at our institution is defined as thresholds of ≤ 30 dBnHL from 0.5 kHz -1 kHz and ≤ 20 dBnHL at 2 kHz - 8 kHz, using both earphone and inserts as transducers.

All evaluable baseline hearing tests required at least 2 thresholds from 1 kHz - 4 kHz. If any frequency was outside the defined normal range, from 1kHz - 4kHz, the audiogram for that ear was not evaluable. Ears with conductive hearing loss ≥ 1000 Hz at baseline were excluded. Patients were included in our analysis if they presented with normal hearing in at least one ear at baseline. In this case, audiologic data was only collected on the single, normal hearing ear. Finally, our study allowed the baseline audiogram to be absent if a subsequent audiogram documented normal hearing.

### Most Recent Audiograms

All audiograms evaluated as the “most recent audiogram” were behavioral hearing tests; soundfield alone and ABR testing were not permissible. All sensorineural hearing loss ≤ 4 kHz, were confirmed by bone conduction thresholds. Bone is not tested above 4 kHz at our institution. If the hearing loss was observed at ≥ 6 kHz, documentation of a normal middle ear status through static admittance of ≥ 0.3 mmho, large ear canal volume consistent with patent tubes, and/or a normal otologic exam by an otolaryngologist was needed to confirm sensorineural nature of the hearing loss. In the current study, the presence of normal bone conduction thresholds and/or a normal hearing evaluation at both baseline and at the most recent hearing test superseded abnormal tympanometric measures. The patient was determined to have hearing loss if the most recent audiogram showed change from normal baseline and a SIOP grade of greater than 0 in the worse evaluable ear.

Each evaluable ear was assigned a SIOP grade relative to the bone conduction thresholds of the most recent audiogram. The SIOP grading scale has been suggested as the superior grading scale in regards to classifying ototoxic hearing loss (17, 18). Audiograms with a normal 4 kHz threshold but an absent or an unevaluable 6 kHz and 8 kHz threshold were codified as “not gradable test”, meaning that it could not utilize the SIOP classification. For this study, SIOP grades 1 and 3 were based on at least one obtained frequency referenced in the SIOP grade level (6 or 8 kHz in grade 1 and 2 or 3 kHz in grade 3). Only 4 patients with hearing loss were not assigned a SIOP grade, as they were still undergoing treatment. Assignment of a SIOP grade to a patient with ongoing treatment would not accurately represent the total ototoxic damage that may develop once the patient’s CDDP treatment is completed.

### Statistical Methods

Study data were collected and managed using REDCap electronic data capture tools hosted at Washington University School of Medicine (19, 20). REDCap (Research Electronic Data Capture) is a secure, web-based software platform designed to support data capture for research studies, providing 1) an intuitive interface for validated data capture; 2) audit trails for tracking data manipulation and export procedures; 3) automated export procedures for seamless data downloads to common statistical packages; and 4) procedures for data integration and interoperability with external sources

All statistical analyses were carried out using SAS version 9.4 (SAS Institute, Cary, NC). Demographic and clinical characteristics were summarized by descriptive statistics, i.e., median and IQR (interquartile range) for continuous variables; and count and percentage for categorical variables. Group differences were examined by Kruskal-Wallis test for continuous variables and Fisher’s exact test (if cell count less than 5) or Chi-square test for categorical variables. Univariate and multivariate logistic regression were used to examine the relationship between hearing loss/SIOP grade and various risk factors, including sex, race, diagnosis, carboplatin use, radiation, radiation to the head, age, total number of doses, the amount of CDDP infused per dose, number of doses/cycle, number of cycles, prescribed dose time, and cumulative CDDP dose. A stepwise variable selection procedure was performed in the multivariate model building for the outcome measure, hearing loss. A significance level of 0.3 was required to allow a risk factor to enter or stay in a model during the variable selection process. To make the results comparable, the final multivariate risk model of hearing loss that obtained during the stepwise selection was used as the multivariate risk model for the outcome measure, SIOP grade.

## Results

**Figure 1:**
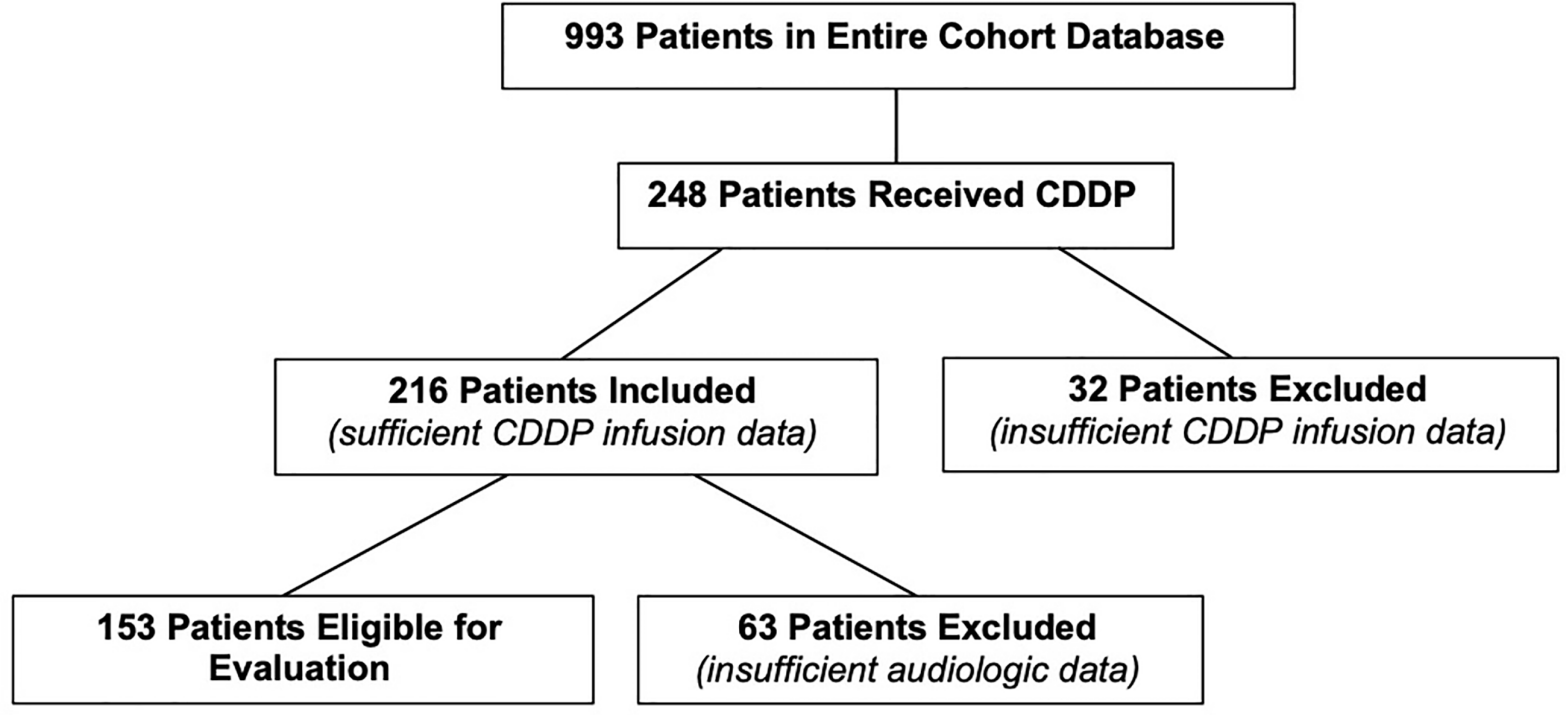
CONSORT diagram outlining selection of eligible patients.

There were 993 patients in the entire cohort. After assessing their treatment, 248 patients were included in the study who had a prior history of CDDP exposure (**Figure 1**). Thirty-two patients were excluded as specific CDDP dosage information was not available. This resulted in a total of 216 evaluable patients for the current study. Of the total 216 patients, 153 had sufficient audiometric data necessary to assess their hearing status.

**Table 1** summarizes the patient and treatment characteristics based on the presence or absence of ototoxic hearing loss. The demographics of patients included in this study are representative of the general pediatric cancer population treated at St. Louis Children’s Hospital – predominately Caucasian with a slight male predominance. The diagnoses of patients in this study reflect patient populations that undergo treatment regimens containing CDDP. There were 111 patients (72.55%) with hearing loss, and 42 (27.45%) with normal hearing. Eight-five (55.6%) of patients were male, and 68 (44.4%) were female. The average age of our patient population was 8.3 years old, ranging from 0.2 to 19.9 years of age. The mean time in years from the last CDDP administration to the last recorded audiogram was 3.73 years (standard deviation= ± 3.77 years). Thirty-one patients (20.3%) were treated with carboplatin in addition to CDDP. There were 91 patients (59.5%) who received radiation, with 65 patients (42.5%) receiving radiation to the head. The mean CDDP cumulative dose of 391.2 mg/m ^2^, ranging from 90 to 1000 mg/m ^2^. The mean amount per dose was 74.1 mg/m ^2^, ranging from 20 to 150 mg/m ^2^. Patients received an average of 6.9 doses (range=1-30) and 3.7 cycles (range=1-8) with an average of 2.1 dose per cycle (range= 1-9). The average prescribed dose time was 4.4 hours, (range= 1-8).

**Table 1:** Patient and treatment characteristics by the presence of hearing loss.

Characteristics^b^	Presence of Hearing Loss^a^			
	Total	Yes	No	*p* ^c^
**No. of Patient**	153 (100%)	111 (72.55%)	42 (27.45%)	
**SIOP Grade**
0	41 (27.9%)	0 (0%)	41 (100%)	
1-2	55 (37.4%)	55 (51.9%)	0 (0%)	
3-4	51 (34.7%)	51 (48.1%)	0 (0%)	
**Age (Years)**	153, 7.8 (3-13.3)	111, 6.1 (2.6-11.2)	42, 13.1 (7.2-16.2)	0.0003
**Sex**				0.3949
Male	85 (55.6%)	64 (57.7%)	21 (50%)	
Female	68 (44.4%)	47 (42.3%)	21 (50%)	
**Race**				0.9819
White	126 (83.4%)	91 (83.5%)	35 (83.3%)	
Non-White	25 (16.6%)	18 (16.5%)	7 (16.7%)	
**Diagnosis**				0.0013
Medulloblastoma	42 (27.5%)	35 (31.5%)	7 (16.7%)	
Non- Medulloblastoma brain tumor	21 (13.7%)	18 (16.2%)	3 (7.1%)	
Neuroblastoma	24 (15.7%)	21 (18.9%)	3 (7.1%)	
Osteosarcoma	32 (20.9%)	15 (13.5%)	17 (40.5%)	
Other	34 (22.2%)	22 (19.8%)	12 (28.6%)	
**Carboplatin Use**				0.4962
Yes	31 (20.3%)	24 (21.6%)	7 (16.7%)	
No	122 (79.7%)	87 (78.4%)	35 (83.3%)	
**Radiation**				0.0100
Yes	91 (59.5%)	73 (65.8%)	18 (42.9%)	
No	62 (40.5%)	38 (34.2%)	24 (57.1%)	
**Radiation to the Head**				0.0121
Yes	65 (42.5%)	54 (48.6%)	11 (26.2%)	
No	88 (57.5%)	57 (51.4%)	31 (73.8%)	
**Total Number of Doses**	147, 6 (4-8)	107, 6 (4-8)	40, 6 (4-8)	0.2614
**Amount of CDDP Infused Per Dose (mg/m2)**	147, 75 (50-90)	107, 75 (50-100)	40, 60 (55-75)	0.0193
**Number of Doses/Cycle**	146, 1 (1-3)	106, 1 (1-4)	40, 2 (1-2.5)	0.0482
**Number of Cycles**	146, 4 (2-5)	106, 4 (2-5)	40, 4 (2-4.5)	0.8423
**Prescribed Dose Time (hours)**	122, 6 (4-6)	86, 6 (1.5-6)	36, 4 (4-6)	0.2135
**Cumulative CDDP Dose (mg/m2)**	153, 400 (300-480)	111, 400 (300-480)	42, 383.5 (299.7-480)	0.3272

**^a^**Hearing loss defined by > 0 SIOP grade.

**^b^**For categorical variables, (n (%)) is reported, excluding missing values. For continuous variables, “n, median (Q1-Q3)” is reported, where Q1 is 25^th^ percentile, and Q3 is 75^th^ percentile.

**^c^**Fisher’s exact test (if cell count less than 5) or Chi-square test for categorical variable; Kruskal-Wallis test for continuous variable.

### Hearing Loss

Analysis of evaluable patients revealed a difference in hearing loss frequency linked to age (p=0.0003), diagnosis (p= 0.0013), radiation to any part of the body (p=0.010), radiation to the head (p=0.012), amount of CDDP per dose (p=0.019), and number of doses per cycle (p=0.048) (**Table 1**). Univariate binary logistic regression analyses demonstrated that hearing loss was associated with age (p= 0.0002), diagnosis (p=.0021), radiation to any part of the body (p=0.011), radiation to the head (p=0.014) and amount of CDDP per dose (p=0.01) (**Table 2**). Multivariate binary logistic regression revealed younger age [OR=0.90 (0.84-0.97), p = 0.0086], radiation to any part of the body [OR= 3.2 (1.3-7.9), p=0.012], larger amount of CDDP per dose [OR=1.018 (1.002-1.033), p=0.029], and larger cumulative CDDP dose [OR=1.004 (1-1.008), p=0.027)] significantly increased the risk of hearing loss (**Table 2**).

**Table 2:** Univariate and multivariate binary logistic regression sasessing risk factors for the presence of hearing loss.

Risk Factors	Univariate		Multivariate^a^	
	Odds Ratio (95% CI)	*p*-value	Odds Ratio (95% CI)	*p*-value
**Age (Years)**	0.878 (0.82-0.94)	0.0002	0.902 (0.835-0.974)	0.0086
**Sex**		0.3957		
Female	0.734 (0.36-1.497)			
Male	-ref-			
**Race**		0.9819		
Non-White	0.989 (0.38-2.573)			
White	-ref-			
**Diagnosis**		0.0021		
Medulloblastoma	2.727 (0.932-7.982)			
Non- Medulloblastoma brain tumor	3.273 (0.799-13.407)			
Neuroblastoma	3.818 (0.942-15.471)			
Osteosarcoma	0.481 (0.179-1.293)			
Other	-ref-			
**Carboplatin Use**		0.4975		
Yes	1.379 (0.545-3.492)			
No	-ref-			
**Radiation**		0.0111		0.0122
Yes	2.561 (1.239-5.294)		3.197 (1.289-7.933)	
No	-ref-		-ref-	
**Radiation to the Head**		0.0139		
Yes	2.67 (1.221-5.836)			
No	-ref-			
**Total Number of Doses**	0.954 (0.896-1.015)	0.1380		
**Amount of CDDP Infused Per Dose (mg/m2)**	1.018 (1.004-1.032)	0.0103	1.018 (1.002-1.033)	0.0287
**Number of Doses/Cycle**	0.91 (0.727-1.14)	0.4144		
**Number of Cycles**	1.031 (0.825-1.289)	0.7859		
**Prescribed Dose Time (hours)**	1.08 (0.902-1.294)	0.4000		
**Cumulative CDDP Dose (mg/m2)**	1.002 (0.999-1.004)	0.2153	1.004 (1-1.008)	0.0271

**^a^**Multivariate stepwise selection results, where a significance level of 0.3 was required to allow a risk factor to enter or stay in a model during the variable selection process.

### SIOP Grade

Univariate and multivariate multinomial logistic regression analyses were also completed to determine associated risk factors with SIOP grade (**Table 3**). Forty-one (27.9%) individuals had a SIOP grade of 0, consistent with normal hearing. Fifty-five (37.4%) of patients had a SIOP grade of 1-2, and 51 (34.7%) had a SIOP grade of 3-4. Worsening SIOP grade was associated with age (p<0.0001), diagnosis (p=0.011), radiation to any part of the body (p=0.022), radiation to the head (p=0.042), and amount of CDDP per dose (0.0093) on univariate analysis. Like hearing loss, worsening SIOP grade was associated with age (p <0.0001), radiation to any part of the body (p=0.023), and amount of CDDP per dose (p=0.037) on multivariate analysis, with cumulative dosing trending toward significance (0.057) (**Table 3**).

**Table 3:** Univariate and multivariate multinomial logistic regression assessing risk factors for SIOP grade.

Risk Factor	SIOP^a^					
	Univariate			Multivariate^b^		
	Odds Ratio (95% CI)		*p*	Odds Ratio (95% CI)		*p*
	Grade 1-2	Grade 3-4		Grade 1-2	Grade 3-4	
**Age (Years)**	0.951 (0.881-1.027)	0.765 (0.692-0.846)	<.0001	0.973 (0.89-1.062)	0.78 (0.697-0.872)	<.0001
**Sex**			0.6909			
Female	0.7 (0.31-1.583)	0.797 (0.349-1.819)				
Male	-ref-	-ref-				
**Race**			0.7766			
Non-White	1.13 (0.389-3.278)	0.773 (0.247-2.414)				
White	-ref-	-ref-				
**Diagnosis**			0.0108			
Medulloblastoma	1.978 (0.601-6.514)	3.429 (1.004-11.712)				
Non- Medulloblastoma brain tumor	2.462 (0.527-11.5)	4 (0.835-19.162)				
Neuroblastoma	2.462 (0.527-11.5)	5.778 (1.258-26.526)				
Osteosarcoma	0.635 (0.212-1.902)	0.167 (0.03-0.917)				
Other	-ref-	-ref-				
**Carboplatin Use**			0.5360			
Yes	1.079 (0.373-3.127)	1.662 (0.594-4.649)				
No	-ref-	-ref-				
**Radiation**			0.0223			0.0233
Yes	2.069 (0.909-4.708)	3.377 (1.413-8.069)		3.065 (1.121-8.384)	4.893 (1.437-16.653)	
No	-ref-	-ref-		-ref-	-ref-	
**Radiation to the Head**			0.0420			
Yes	2.273 (0.951-5.431)	3.068 (1.269-7.419)				
No	-ref-	-ref-				
**Total Number of Doses**	0.955 (0.888-1.027)	0.949 (0.88-1.024)	0.2994			
**Amount of CDDP Infused Per Dose (mg/m2)**	1.015 (1-1.031)	1.025 (1.009-1.042)	0.0093	1.019 (1.001-1.037)	1.026 (1.006-1.046)	0.0369
**Number of Doses/Cycle**	0.853 (0.652-1.116)	0.97 (0.753-1.25)	0.4653			
**Number of Cycles**	1.048 (0.815-1.348)	0.96 (0.742-1.241)	0.7674			
**Prescribed Dose Time (hours)**	1.094 (0.894-1.339)	1.055 (0.854-1.304)	0.6837			
**Cumulative CDDP Dose (mg/m2)**	1.002 (0.999-1.005)	1.002 (0.999-1.005)	0.4528	1.005 (1.001-1.009)	1.005 (1-1.009)	0.0565

a for multinomial logistic regression, logits modeled use “SIOP Grade 0” as the reference category.

b to make the results comparable, the final multivariate risk model of hearing loss that obtained during the stepwise selection was used as the multivariate risk model for the outcome measure, SIOP grade.

## Discussion

Increasing our understanding of the parameters of CDDP infusion effects on hearing loss can lead to strategies that may reduce the risk. This study has demonstrated the amount of CDDP infused per dose was strongly associated with an increased risk of hearing loss; it is also associated with more severe hearing loss as reflected by a worsening SIOP grade. Other parameters such as number of doses, number of doses per cycle, number of cycles, and infusion times failed to reach significance using multivariate analysis. Cumulative CDDP was also associated with hearing loss, to a lesser degree. Prior studies have repeatedly identified cumulative incidence as a risk factor for hearing loss (4, 11, 21). The relatively modest impact of cumulative dosing of CDDP and hearing loss in this study may be due to the relative lack of variance in our cohort in their cumulative dosing (25^th^-75^th^ percentile = 300-480 mg/m ^2^). Thus, cumulative dosing may have been obscured as a risk factor due to the minimal difference in cumulative dosing between those with hearing loss and those without (mean = 400 mg/m^2^ *vs.* 383.5 mg/m^2^).

Our analysis also revealed age was strongly associated with a risk of hearing loss with the youngest patients at greatest risk. Radiation to any part of the body was also shown to be a risk factor, with radiation directly to the head failing to achieve significance in multivariate analysis. Our previous investigations had established radiation to locations other than the head still place patients at risk for hearing loss (16). Radiation can lead to circulating free radicals and inflammatory mediators that have been reported to impact organs and tissues distant from the organ targeted for radiation (ascopal effect) (22, 23). It is likely radiation to the head is also a risk factor for hearing loss given the significant correlation observed in univariate analysis. Perhaps a larger patient population would clarify its significance.

This report supports a recent study published which also identified the amount per dose as a risk factor for hearing loss (24). Our study can be distinguished from that report due to the following differences. In the aforementioned study, baseline audiograms were not required for the analysis; therefore there is no assurance whether any of the patients had a pre-existing bilateral or unilateral hearing loss prior to the initiation of CDDP therapy. We continued to utilize the rigorous criteria we have used in previous studies to ensure hearing loss in our subjects was a consequence of acquired sensorineural hearing loss, excluding ambiguous tests results. All of the subjects in our report were tested in the same audiology department, using standardized procedures and workflows with calibrated equipment in sound suites minimizing interinstitutional variance. To be evaluable, audiograms had to be of good to fair reliability with well-defined parameters to exclude patients with pre-existing hearing loss. Because our most recent audiograms required ear specific thresholds for inclusion, we were able to attribute hearing loss on the worse ear capturing the true effects of CDDP dosing on our patients. We routinely utilize the worse ear to designate hearing loss in our patients given the significant affect unilateral hearing loss can have on quality of life (25). Standard of care at our institution includes testing inter-octave thresholds when appropriate which allows for accurate SIOP grading.

Our audiometric data is representative of a purely pediatric population; our mean age is older at 8.3 years. Obtaining reliable data in a young, ill child is fraught with difficulties, leading to audiograms that often fail to meet our stringent criteria for study eligibility. Many audiograms in our youngest subjects were not included in this study driving up the average age of the cohort. Finally, we included hearing loss at all levels of severity, while the recent publication only examined patients with higher SIOP grades. We had previously shown hearing loss continues to decline long after therapy is completed. We also have demonstrated that the patients with the longest follow-up had the highest likelihood of diagnosed hearing loss. Our mean time in years from the last CDDP administration to the last audiogram was 3.73 years while the previous study reported a mean of 1.5 years from the end of treatment. The increased prevalence of hearing loss as patients are followed longer and the use of the better verses worse ear may explain the difference in hearing loss observed by those investigators (43.8%) compared to our study (72.6%) (24).This late onset hearing loss particularly affects patients with established hearing loss at the completion of therapy. Thus, even mild and unilateral CDDP associated hearing loss is significant and warrants further follow-up.

This study was limited by the large fraction of patients (39%) excluded for analysis due to missing infusion or audiology data. .Ineligible patients were younger, more frequently treated with carboplatin, and were less likely to be treated with radiation. We suspect that these findings could be attributed to the differences in the number of neuroblastoma patients in the ineligible cohort (15.7% Eligible yes, 30.5% Eligible no). Neuroblastoma patients are typically younger, may receive carboplatin, and may receive less radiation due to their young age. Given the difficulties in obtaining audiograms in young patients, it is possible that young neuroblastoma patients were disproportionately excluded due to insufficient audiologic data

The study was a cross sectional analysis with no specific time point that could be used for hearing testing that could encompass the entire cohort. Audiograms were not obtained after each CDDP infusion so we could not assess any variables associated with a specific infusion encounter. However, despite these limitations and given the paucity of studies examining this subject, our audiologic monitoring program provided evidence that calls for new approaches for CDDP administration.

In order to increase the number of evaluable patients, we included patients that demonstrated SNHL, even if they had not completed therapy. However, we did not include patients undergoing therapy if they had not demonstrated hearing loss at the end of the study period. Given that our patients were carefully screened for SNHL, which we would expect to be irreversible, we were not concerned that patients who had established SNHL would subsequently convert to the “no hearing loss” cohort with time. In contrast, there are patients who may lose hearing with subsequent cycles; this was our justification for not including those patients receiving therapy in the no hearing loss group. Furthermore, the patients with hearing loss on therapy were not included in the SIOP analysis, as it is possible the SIOP grade could shift, even while on therapy. We therefore were able to add additional patients to enhance our sample size, without including those subjects that would compromise the integrity of the cohort.

This study also collected patients over a period of almost thirty years. Although there have been advances in this field, there are no significant changes in audiology testing that would have influenced the data. However our monitoring of patients became more robust in recent years, where we now test at risk patients at regular and set intervals. Thus, the results may have been influence by the frequency of testing rather than differences in testing methods. We addressed this issue by examining the year of diagnosis (based on the date of diagnosis) as a continuous variable. The year of date of diagnosis ranges from 1983 to 2015 (median: 2006). The median diagnosis year of patients with hearing loss is 2005 ranging from 1983 to 2015; while the median diagnosis year of non-hearing loss group is 2007 ranging from 1996 to 2013. There is no significant relationship between hearing loss and the year of diagnosis to the date of the last audiogram, [OR = 0.96 (0.90- 1.03), p- = 0.22)]. Subgroup analysis comparing 2010-2015 to 1990-1995 also shows the year of diagnosis has no significant relationship with hearing loss (p = 0.16).

Although we examined specific infusion parameters, we were unable to assess how patients may vary in CDDP exposure based on varying pharmacokinetics. Such variations could lead to variances in area underneath the curve (AUC) that could account for different levels of toxicity. Calvert established a formula to establish more consistent dosing and AUC attainment for carboplatin (26). Unfortunately, due to the unpredictable pharmacokinetics of cisplatin, comparable formulas have yet to be developed (18, 27). More research is needed in this field.

Despite these limitations and given the paucity of studies examining this subject, our audiologic monitoring program provided evidence that calls for new approaches for CDDP administration.

This project is one of the largest audiologic studies examining the influence of CDDP dosing and schedules on hearing loss. We demonstrated the amount of CDDP infused per dose is a significant risk factor. These findings support an observation in a previously reported clinical trial by the Children’s Oncology Group where the two cohorts only varied with the amount of CDDP per dose with worse ototoxicity observed in the group assigned the higher dose (13). Such dosing can lead to higher peak serum levels that can lead to greater penetration into the cochlea. Given the recent studies demonstrating the persistent retention of CDDP in the cochlea, alternative dosing with lower amounts per dose may reduce CDDP accumulation in the cochlea and may potentially lead to less ototoxicity while retaining its anti-neoplastic properties. Prospective clinical trials are needed.

## Data Availability Statement

The original contributions presented in the study are included in the supplementary material. Further inquiries can be directed to the corresponding authors.

## Ethics Statement

The studies involving human participants were reviewed and approved by Institutional Review Board of the Washington University School of Medicine Human Research Protection Office. Written informed consent from the participants’ legal guardian/next of kin was not required to participate in this study in accordance with the national legislation and the institutional requirements.

## Author Contributions 

Design of the trial – RH, SH, Execution of the trial – RH, SH, JH, KS, Data collection – SH, MC, AS, Data analysis – NW, Writing Manuscript – RH, SH, MC, AS, NW. All authors contributed to the article and approved the submitted version.

## Funding

Data analysis was provided by the Biostatistics Shared Resource, of The Siteman Cancer Center and is supported in part by an NCI Cancer Center Support Grant #P30 CA091842, Eberlein, PI.

## Conflict of Interest

The authors declare that the research was conducted in the absence of any commercial or financial relationships that could be construed as a potential conflict of interest.
